# Cascade Electric Field in ZnIn_2_S_4_/CuCo_2_O_4_ Photocatalyst for the Selective Oxidation of Biomass‐Derived 5‐Hydroxymethylfurfural in Aqueous Solutions

**DOI:** 10.1002/smll.202409005

**Published:** 2025-03-19

**Authors:** Yixuan Liu, Wenhua Xue, Jian Ye, Ruilong Zhang, Akkammagari Putta Rangappa, Jun Zhao

**Affiliations:** ^1^ Sino‐Forest Applied Research Centre for Pearl River Delta Environment Department of Biology Hong Kong Baptist University Kowloon Tong Hong Kong SAR 999077 China; ^2^ Institute of Advanced Materials Hong Kong Baptist University Kowloon Tong Hong Kong SAR 999077 China

**Keywords:** 5‐hydroxymethylfurfural, biomass, CuCo_2_O_4_, photocatalysis, ZnIn_2_S_4_

## Abstract

The photocatalytic conversion of biomass feedstock represents a promising and environmentally friendly strategy for achieving selective transformation and value addition. The slow charge dynamics and sluggish hole transfer in the oxidation reactions severely limit the photocatalytic activity. Here, the heterojunction is fabricated by synthesizing ultra thin ZnIn_2_S_4_ nanoflower with spinel CuCo_2_O_4_. The internal and interfacial electric fields are successfully constructed, which shows superior 5‐hydroxymethylfurfural (HMF) valorization. HMF undergoes severe mineralization when ZnIn_2_S_4_ is used as the catalyst, resulting in 0.9% 2,5‐diformylfuran (DFF) yield in water, while the ZnIn_2_S_4_/CuCo_2_O_4_ heterojunction catalyst exhibits 77% DFF selectivity with 88.6% HMF conversion, The cascaded bulk and internal electric fields greatly reduce the oxidation potential of holes and enhance the charge separation efficiency, thus give a remarkable 70‐fold increase in DFF yield. This work overcomes the limitations of ZnIn_2_S_4_ application for HMF and similar alcohol oxidation reactions that typically require organic solvents, achieving a high DFF evolution rate of 724.9 µmol·g^−1^·h^−1^ in water within the first hour of the reaction, surpassing most reports of photocatalytic HMF selective oxidation.

## Graphical abstract

The internal and interfacial electric fields are constructed in ZnIn_2_S_4_/10%CuCo_2_O_4_ by synthesizing ZnIn_2_S_4_ nanoflower with CuCo_2_O_4,_ which shows a remarkable 70‐fold increase in DFF yield than ZnIn_2_S_4_ for HMF oxidation in water. The cascaded electric fields greatly reduce the oxidation potential of holes and overcome the limitations of ZnIn_2_S_4_ application for HMF and similar alcohol oxidation reactions that typically require organic solvents.

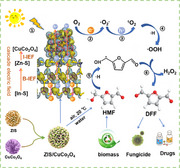

## 1 Introduction

The abundant storage of biomass is considered a green, renewable, and sustainable resource. Significant efforts have been made to explore effective strategies for converting biomass from waste to treasure.^[^ ^1^ ^]^ 5‐Hydroxymethylfurfural (HMF) is a common building‐block chemical derived from biomass, possessing active functional groups (furan ring, alcohol hydroxyl group, aldehyde group).^[^ ^2^ ^]^ These functional groups make it advantageous for generating value‐added products like 2,5‐diformylfuran (DFF), a compound extensively utilized in pharmaceuticals and synthetic resin production.

Solar‐driven photocatalytic methods are regarded as environmentally friendly and energy‐saving processes, making the utilization of semiconductor photocatalysis an ideal technology for addressing biomass conversion.^[^ ^3^ ^]^ Among various materials,^[^ ^4^ ^]^ ZnIn_2_S_4_ (ZIS), with its ultra thin 2D structure, has attracted widespread attention due to its mild valance potential, high reduction potential, and outstanding photostability.^[^ ^5^ ^]^ Based on ZIS, many efforts have been proposed for the selective oxidation of HMF. For instance, O‐doped ZIS nanosheets,^[^ ^6^ ^]^ ZIS nanoparticles with Zn vacancies.^[^ ^7^ ^]^ S‐vacancy‐Rich Zn_3_In_2_S_6_/Bi_2_MoO_6_.^[^ ^8^ ^]^ However, most existing catalysts work only in organic solvent,^[^ ^9^ ^]^ which not only increases the cost of the reaction process but also brings extra environmental risks due to the utilization of organic solvents like acetonitrile (ACN), tetrahydrofuran (THF), and DMSO et.^[^ ^10^ ^]^ In addition, the low product evolution (NiS/Zn_3_In_2_S_6_ for 129 µmol·g^−1^·h^−1^, ZnIn_2_S_4_/Nb_2_O_5_ for 251 µmol·g^−1^·h^−1^) also presents a challenging issue for the practical application.^[^ ^11^ ^]^

Generally, when water is used as the solvent, the strong oxidation active species will cause HMF mineralization, thus leading to very low DFF selectivity and yield. This phenomenon can be principally attributed to two underlying reasons: 1) The strong polarity of water substantially enhances the reactivity of radical species, leading to the mineralization of HMF; 2) The tunable yet suboptimal oxidative power of holes results in a non‐negligible contribution of hole‐induced HMF degradation across most systems, which is particularly pronounced in the case of g‐C_3_N_4_ and TiO_2_ based catalysts. Additionally, the rapid recombination of photogenerated electrons (e^−^) and holes (h^+^) within picosecond timescales also significantly surpasses their migration to catalytic sites (≈hundreds of picoseconds) and participation in surface catalytic reactions (≈nanoseconds to milliseconds). Thus, the sluggish charge kinetics results in low surface reaction efficiency and catalytic activity.^[^ ^12^ ^]^

To mitigate the aforementioned challenges, the implementation of an internal electric field (IEF) has been proposed as a means to enhance photocatalytic activity. The approach involves constructing a bulk IEF (B‐IEF) within the catalyst by exploiting asymmetrical crystal polarities and combining with another material to create a surface heterojunction structure with nonuniform charge distribution. This strategy not only enables the formation of a cascading electric field to enhance the separation of charges in opposite directions but also reduces the oxidation potential of photoinduced holes to avoid the overoxidation of the reactant and lessen the non‐selective radicals in aqueous solution. For instance, Wan et al. reported that Ni_12_P_5_/ZnIn_2_S_4_‐O material was used to construct the cascade B‐IEF and interface‐internal electric field (I‐IEF) by O‐doping and ohmic junction.^[^ ^13^ ^]^ This collaborative design can improve the performance of photocatalytic reduction‐oxidation reactions. Inspired by these reports, we are trying to find an appropriate co‐catalyst for ZIS to construct the cascade B‐IEF and I‐IEF for the HMF oxidation, which can facilitate the transfer of surface charge carriers from ZIS to another. Among various materials, spinel‐type transition bimetallic oxides MCo_2_O_4_ may be a suitable candidate due to its excellent chem‐physical stability and mild oxidation potential.^[^ ^14^ ^]^ The *p*‐type semiconductor MCo_2_O_4_ and the *n*‐type semiconductor ZIS possess distinct band gaps. The combination of the two materials is likely to result in the formation of IEF. MCo_2_O_4_ was reported to be used in various photocatalytic applications, like H_2_ evolution,^[^ ^15^ ^]^ CO_2_ reduction,^[^ ^16^ ^]^ and degradation of toxic dyes.^[^ ^17^ ^]^ Nevertheless, as far as we know, the potential of spinel in photocatalytic biomass conversion has not been explored.

In this study, we proposed a strategy to enhance charge separation and migration in the ZIS photocatalyst by introducing CuCo_2_O_4_ to construct cascaded electric fields. The incorporation of CuCo_2_O_4_ has significantly improved carrier separation efficiency and surface reaction kinetics while also reducing the oxidative potential of holes to suppress HMF mineralization. This approach has demonstrated exceptional performance, achieving 87.6% conversion of HMF and a 63.0% yield of DFF in aqueous media, surpassing previously reported results (see Table S1, Supporting Information). Both experimental and theoretical calculations were used to verify the reaction mechanism and the role of various radicals in this oxidation process. This work lays a foundation for extending similar strategies to build efficient photocatalytic biomass valorization systems in aqueous media.

## 2 Results and Discussion

**Figure 1:**
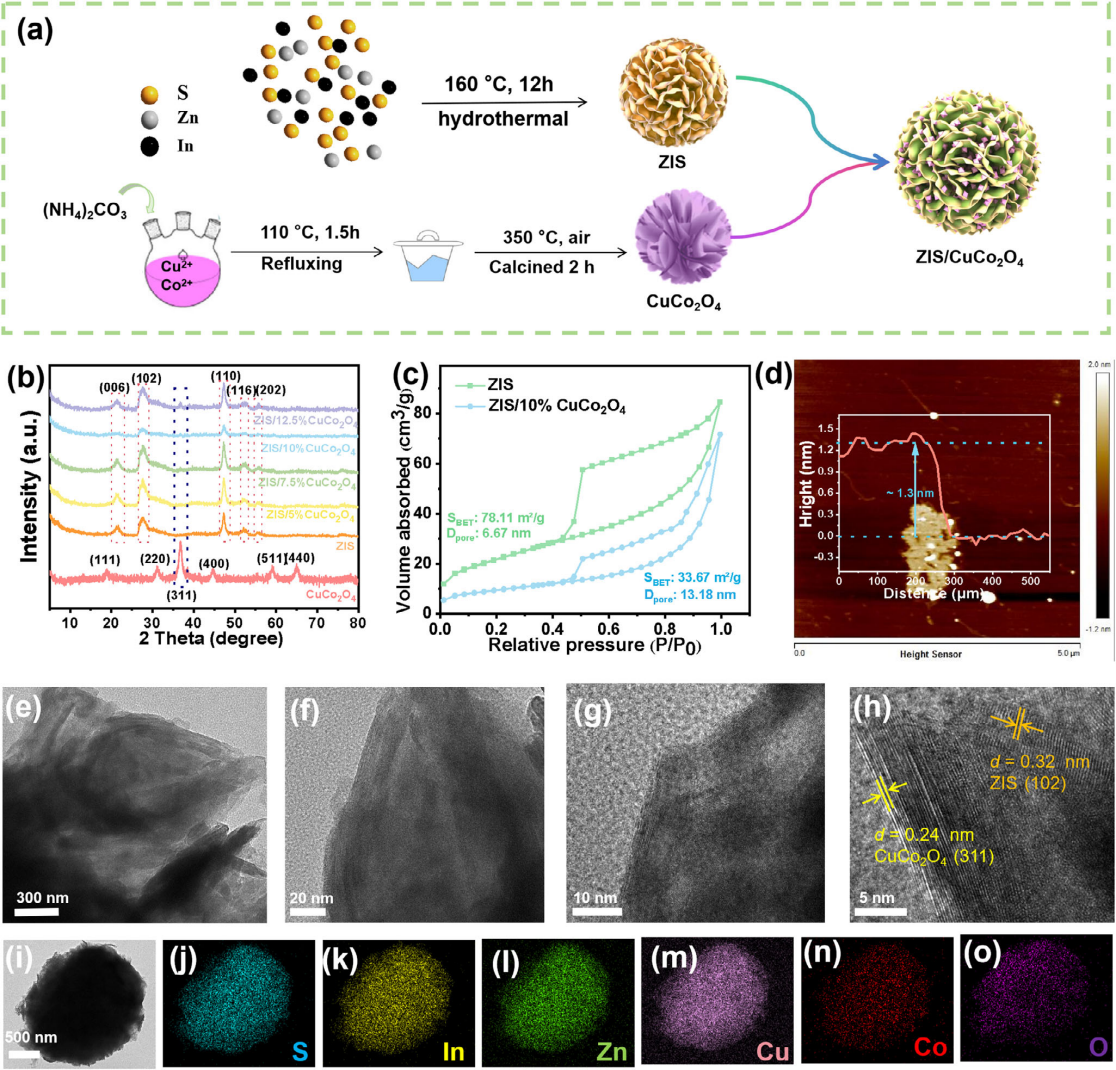
a) Schematic process for the synthesis of ZIS/CuCo_2_O_4_. b) XRD, c) N_2_ adsorption/desorption isotherms. d) 2D AFM image of ZIS. e–h) TEM images and i–o) Elemental Mapping of ZIS/10%CuCo_2_O_4_.

As illustrated in **Figure** **1**a, ZIS can be readily synthesized via a hydrothermal method, while CuCo_2_O_4_ requires two steps of hydrothermal treatment and calcination during preparation. Ultimately, the two components are added in different proportions to ethanol and processed by a drying method to obtain ZIS/CuCo_2_O_4_ heterostructure materials.

The X‐ray diffraction (XRD) patterns of different ZIS/CuCo_2_O_4_ are presented in Figure 1b. Due to the small amount of addition of CuCo_2_O_4_, different ZIS/CuCo_2_O_4_ displayed similar characteristic peaks and hexagonal crystalline structures with ZIS (JCPDS No. 72–0773). The 2‐theta values at 21.6°, 27.7°, 47.2°, 52.4° and 55.6° corresponded to the diffraction of the (006), (102), (110), (116) and (202) crystal planes of ZIS, respectively. The peaks located at 19.07°, 31.36°, 36.96°, 45.01°, 59.60°, and 65.70° belonged to the (111), (220), (311), (400), (511), and (440) crystal planes of CuCo_2_O_4_, respectively, which were well matched with the standard diffraction pattern (JCPDS No. 01–1155). This result proved that we successfully synthesized ZIS and CuCo_2_O_4_ without other impurities. Compared to ZIS, ZIS/CuCo_2_O_4_ with different CuCo_2_O_4_ contents showed no significant changes, indicating that introducing CuCo_2_O_4_ did not alter the phase and crystallinity of ZIS. However, with increasing CuCo_2_O_4_ content, the characteristic peak of 36.96° appears in ZIS/CuCo_2_O_4_, indicating the successful formation of the ZIS/CuCo_2_O_4_ heterojunction. Moreover, N_2_ absorption‐desorption curves suggested the specific surface area (S_BET_) of ZIS and ZIS/10%CuCo_2_O_4_ 78.11 and 33.67 m^2^ g^−1^, respectively in Figure 1c. The successful combination of ZIS thin layers in two dimensions could lead to a certain degree of stacking and adhesion under the modification of CuCo_2_O_4_, resulting in a reduction in specific surface area. The atomic force microscope (AFM) image in Figure 1d indicates that the thinness is ≈1.3 nm. A typical ZIS nanoflower shape was clearly observed from scanning electron microscopy (SEM) (Figure S1a,b, Supporting Information), and the smaller CuCo_2_O_4_ (Figure S1c, Supporting Information) was located on the ultra thin nanolayer of ZIS to construct the ZIS/10%CuCo_2_O_4_ (Figure S1d, Supporting Information). The different lattice spacing of 0.32 and 0.24 nm in the transmission electron microscope (TEM) images attributed to the (102) plane of ZIS and the (311) plane of CuCo_2_O_4_ (Figure 1e–h). Besides, the uniform element (S, In, Zn, Cu, Co, O) distribution (Figure 1i–o) from elemental mapping of ZIS/10%CuCo_2_O_4_ implied well‐dispersed active sites, which potentially favored electronic transport pathways and surface reaction.^[^ ^18^ ^]^

**Figure 2:**
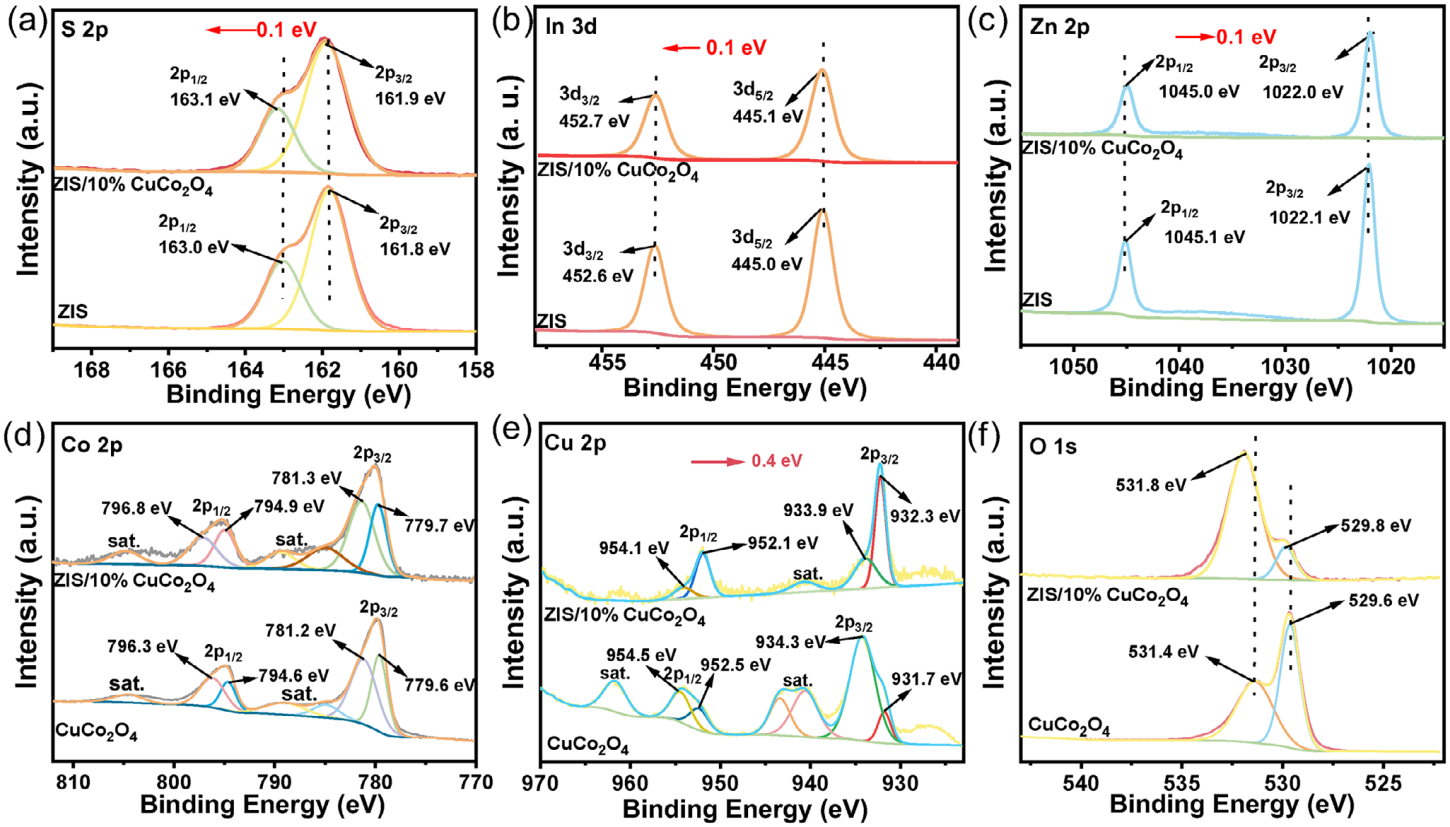
XPS spectra of ZIS, ZIS/10%CuCo_2_O_4,_ and CuCo_2_O_4_ catalysts. a) S 2p, b) In 3d, c) Zn 2p, d) Co 2p, e) Cu 2p, f) O1s.

X‐ray photoelectron spectroscopy (XPS) gives the chemical states and composition of bare ZIS, CuCo_2_O_4,_ and ZIS/10%CuCo_2_O_4_. The survey spectrum of ZIS/10%CuCo_2_O_4_ confirmed the purity of the composites (Figure S2, Supporting Information). The S 2p orbital's binding energy displayed two distinct peaks at 161.8 and 163.0 eV (**Figure** **2**a), corresponding to the S 2p_3/2_ and S 2p_1/2_, respectively.^[^ ^19^ ^]^ Figure 2b,c exhibits individual peaks for In 3d (452.6 and 445.0 eV) and Zn 2p (1045.1 and 1022.1 eV). Compared to pristine ZIS, a shift to higher binding energies for S 2p and In 3d (≈0.1 eV) in the composite was observed, while the Zn 2p moved to lower binding energy. The possible reasons for their opposite shift direction are as follows. An interfacial charge transfer channel of In─S─Cu and Zn─S─Cu was formed between ZIS and CuCo_2_O_4_. In this case, the electron density around the S atom will increase due to the increased electron supply. Noted that the electronegativity of In^3+^ is much lower than that of Zn^2+^ and Cu^2+^ (Zn^2^⁺ ≈ Cu^2^⁺ > In^3^⁺), resulting in S^2−^ being more strongly attracted in the In─S─Cu bond upon the combination of ZIS and CoCu_2_O_4_. This causes the electron cloud density to shift away from In, leading to a leftward shift in the XPS binding energy of In. Conversely, since the electronegativities of Zn^2^⁺ and Cu^2^⁺ are quite similar, the electron cloud density around Zn^2^⁺ will increase in the Zn─S─Cu configuration due to more electron supply by Cu. Regarding Co 2p of CoCu_2_O_4_ (Figure 2d), the spectrum could be deconvoluted into four peaks indicative of Co 2p_3/2_ and Co 2p_1/2_, along with two shake‐up satellite peaks marked as “sat.” The signals at 779.6 and 794.6 eV were indicative of Co^3+^, while the binding energies at 781.2 and 796.3 eV corresponded to Co^2+^.^[^ ^20^ ^]^ Evidently, the Cu^+^ (931.7 and 952.5 eV) and Cu^2+^ (934.3 and 954.5 eV) cations are present in Figure 2e, with two distinct satellite peaks. Following the refined deconvolution of O 1s of CuCo_2_O_4_ (Figure 2f), the two signals at 529.6 and 534.1 eV corresponded to metal‐oxygen bonds (Cu─O and Co─O) and a multitude of oxygen vacancies (defect sites) with O_2_ coordination in CuCo_2_O_4_, respectively. The presence of Cu^+^ and Co^2+^ resulted from the valence change of partial cations caused by O vacancies, ubiquitous in metal oxides.^[^ ^21^ ^]^ It should be noted that the Cu^+^ content increased while the Cu^2+^ content decreased after forming the composite of CuCo_2_O_4_ and ZIS, a phenomenon attributed to electron transfer from ZIS to CuCo_2_O_4_. It is also noteworthy that there was a clear shift to lower binding energies for Cu 2p peaks (≈0.4 eV) in the composite compared with pristine CuCo_2_O_4_. Thus, it is inferred that a Cu─S─In or Cu─S─Zn interfacial chemical bond could be formed, which can act as electron channels for charge transfer.

**Figure 3:**
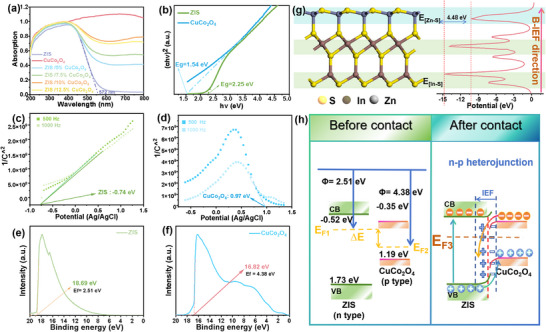
a) UV–vis spectra. b) Kubelka–Munk plots. M‐S curves c) ZIS and d) CuCo_2_O_4_ under different frequencies at pH 7. UPS spectra of e) ZIS and f) CuCo_2_O_4_. (g) DFT‐calculated illustration of the crystal structure and electrostatic potential along the [001] axis for ZIS. h) illustration of the band structures of ZIS and CuCo_2_O_4_ and the formation of the I‐IEF through band bending and the charge transfer pathway at the interface of ZIS/CuCo_2_O_4_.

It is well‐established that the direction and intensity of the induced electric field are closely associated with the energy band structure of the catalysts. UV–visabsorption spectrum, and Mott–Schottky (M–S) measurements of ZIS and ZIS/10%CuCo_2_O_4_ catalyst were utilized to offer more insights. UV–vis spectra (**Figure** **3**a) illustrated that the absorption edge of ZIS was ≈572 nm, while CuCo_2_O_4_ exhibited strong light absorption throughout the ultraviolet to visible light range. It was conceivable that the light absorption of ZIS/CuCo_2_O_4_ also increased with increasing amounts of CuCo_2_O_4_ added. The corresponding Tauc curves (Figure 3b) certified that the bandgap gap (E_g_) of ZIS and CuCo_2_O_4_ were 2.25 and 1.54 eV, respectively. The Mott‐Schottky (MS) curve indicated that ZIS is *n*‐type while CuCo_2_O_4_ is a *p*‐type semiconductor (Figure 3c,d). Accordingly, the conduction band (CB) position of ZIS is −0.74 + 0.22 = −0.52 V due to the Ag/AgCl electrode being used. The measured valence band (VB) position of CuCo_2_O_4_ is 0.97 eV + 0.22 = 1.19 eV, leading to a CB position of 1.19 – 1.54 = −0.35 eV. It is noted that the Fermi level difference between ZIS and CuCo_2_O_4_ is as large as 1.87 eV (2.51 eV for ZIS and 4.38 eV for CuCo_2_O_4_), which is shown in Figure 3e,f. Therefore, the CB of CuCo_2_O_4_ will shift upwards upon contact with ZIS. However, it is hard to determine their specific conduction band and valence band potential after shift. We inferred that the CB potential of CuCo_2_O_4_ may become more negative than that of ZIS, resulting in electron flow from CuCo_2_O_4_ to ZIS, which can be further reflected by later in situ XPS results. Additionally, as mentioned before, the DFT calculation further certified the presence of an intrinsic polarized B‐IEF in pristine ZIS. The ZIS nanosheets exhibited an asymmetric electrostatic potential distribution, with the vacuum level of the [Zn‐S] surface being higher than that of the [In‐S] surface. This arrangement leads to a spontaneous polarization from the [Zn‐S] layer toward the [In‐S] layer at the termini of the two surfaces. The magnitude of the polarization‐induced electric field effect was assessed by measuring the electrostatic potential difference (ΔE). Due to the asymmetrical layered structure of [S–In]–[S–In–S]–[Zn–S] unit cell in ZIS, the IEF existed between [S‐In] and [S‐Zn] layer along the [001] direction (Figure 3g). The asymmetrical crystal structure revealed the nonuniform charge distribution and exhibited the local ∆E of 4.48 eV. These results suggested that a cascaded field had been successfully constructed. Figure 3h shows the band alignment and band bending of ZIS and CuCo_2_O_4_ before and after contacting, where the electrons flow from the CB of CuCo_2_O_4_ to the CB of ZIS under light irradiation driven by an interfacial field. The downward band bending of *p*‐type CuCo_2_O_4_ and upward bending of *n*‐type ZIS creates a “charge‐carriers confinement” effect.^[^ ^22^ ^]^ ZIS photogenerated e^−^ are confined at the high‐reduction‐potential CB due to energy barriers, while h^+^ efficiently transfers through the CuCo_2_O_4_ VB. This mechanism overcomes carrier recombination limitations in conventional type‐I junctions, complementing rather than contradicting classical theory. The lower VB position of ZIS implies that the excessively strong oxidative capacity of its holes would lead to HMF degradation. However, the introduced CuCo_2_O_4_ can transfer these holes to reduce the oxidation ability of h^+^, while e^−^ migrates into the interfacial depletion region, ultimately neutralizing the positive charges at the interface. This mechanism precisely explains the primary scientific issues addressed in this study, that is, nearly complete degradation of HMF occurs when ZIS is solely employed as a catalyst in aqueous solution, whereas a significantly higher DFF yield is achieved using the ZIS/ CuCo_2_O_4_ catalyst.

**Figure 4:**
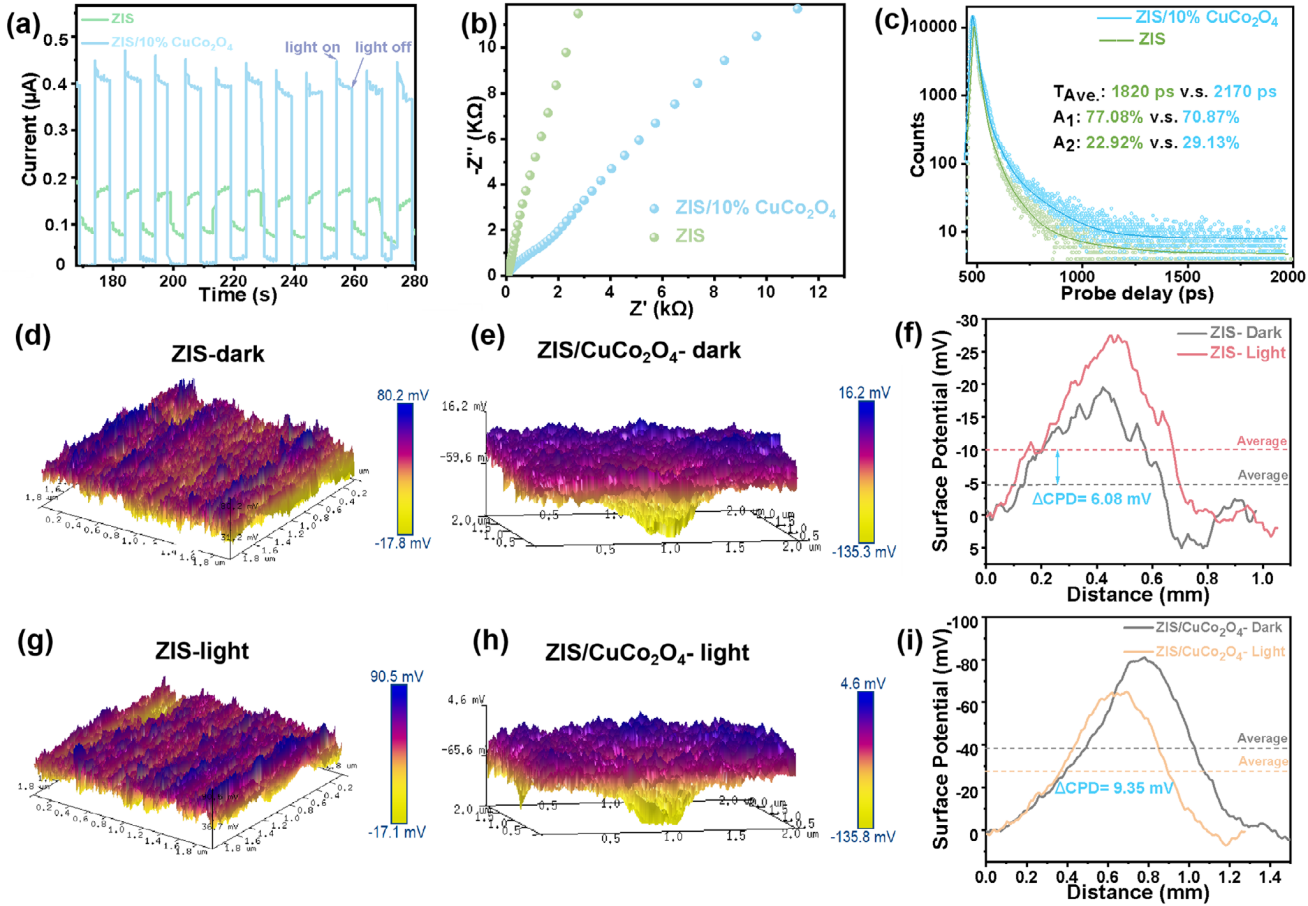
a) Transient photocurrent response spectra. b) EIS spectra. c) transient PL of ZIS and ZIS/10%CuCo_2_O_4_. KPFM potential 3D images along the line in the dark d,e) and under illumination g,h). The corresponding surface potential curves for f) ZIS and i) ZIS/CuCo_2_O_4_.

A series of photoelectrochemical and spectroscopic analyses were then conducted to prove the efficient cascaded internal electron field. Evidently, the transient photocurrent response (**Figure** **4**a) of ZIS/10% CuCo_2_O_4_ (0.56 µA) is significantly higher than that of ZIS (0.13 µA), reaching a value of 4.31 times, indicating a substantial enhancement in the separation and migration efficiency of photo‐generated charge carriers after the introduction of I‐IEF. Moreover, the Nyquist plot (Figure 4b) obtained from Electrochemical Impedance Spectroscopy (EIS) revealed that the ZIS/10%CuCo_2_O_4_ showed a smaller arc radius than ZIS. The relative sizes of the arc radii corresponded to the magnitude of charge transfer resistance and the efficiency of separating photo‐generated electron–hole pairs, the result indicated that the coordination of the cascade electric field was beneficial for the transport of charge carriers. Photoluminescence (PL) spectra (Figure S3, Supporting Information) prove that the emission peak intensity of pristine ZIS is 2.79 times higher compared to the ZIS/10%CuCo_2_O_4_. The sharp quenching of the PL signal indicated that CuCo_2_O_4_ played a significant role in suppressing the recombination of photogenerated charge carriers. In Figure 4c, the transient PL decay kinetics fitting results suggested that the electron lifetime of ZIS/10%CuCo_2_O_4_ (2170 ps) was longer than that of pristine ZIS (1820 ps), attributed to the efficient cascaded field.

To gain a deeper understanding of the charge behavior within a cascading electric field, Kelvin probe force microscopy (KPFM) was employed to measure the contact potential difference (CPD) and surface photovoltage (SPV) after loading CuCo_2_O_4_. An increase in ∆CPD signified a rise in the sample's potential, as the potential of the tip remained constant. The corresponding surface potential curves for ZIS and ZIS/CuCo_2_O_4_ KPFM potential 2D images (Figure S4, Supporting information) and 3D images clearly revealed the CPD variations of pristine ZIS and the composite under dark (Figure 4d,e) and illumination (Figure 4g,h). A significantly higher ∆CPD for ZIS/CuCo_2_O_4_ in the dark was observed, indicating that the composite material facilitated a stronger surface potential. Under light irradiation, the enhanced potential of ZIS/CuCo_2_O_4_ (9.35 mV for composite vs 6.08 mV for pristine ZIS) suggested that CuCo_2_O_4_ was effective in extracting photogenerated h^+^ from the bulk ZIS to the surface (Figure 4f,i).^[^ ^23^ ^]^ Modulated SPV measurements (Figure S5 Supporting Information) display a variation consistent with KPFM results. For an *n*‐type of semiconductor, electrons are captured by surface states under irradiation, resulting in an upward band bending. Simultaneously, due to the net negative charge at the surface and the net positive charge in the space charge region (SCR), a built‐in electric field is established in the SCR, directed from the bulk to the surface.^[^ ^24^ ^]^ Consequently, the surface potential is lower than the potential in the bulk, forming a surface barrier. Thus, the positive SPV signal observed in ZIS indicated the migration of photogenerated e^−^ to the bulk and photogenerated h^+^ to the surface. Notably, the SPV intensity of ZIS/10%CuCo_2_O_4_ was lower than that of ZIS due to the influence of CuCo_2_O_4_, which indicated the h^+^ were transferred from ZIS to CuCo_2_O_4_. In detail, since CuCo_2_O_4_ was deposited on the ZIS surface, the accumulation of photogenerated e^−^ on the interface could counteract the positive photoelectric signal during SPV analysis, leading to a decreased SPV signal in ZIS/10%CuCo_2_O_4_. The modified migration pathway of photogenerated charge carriers enhances the functionality of IEF to facilitate charge kinetics.

**Figure 5:**
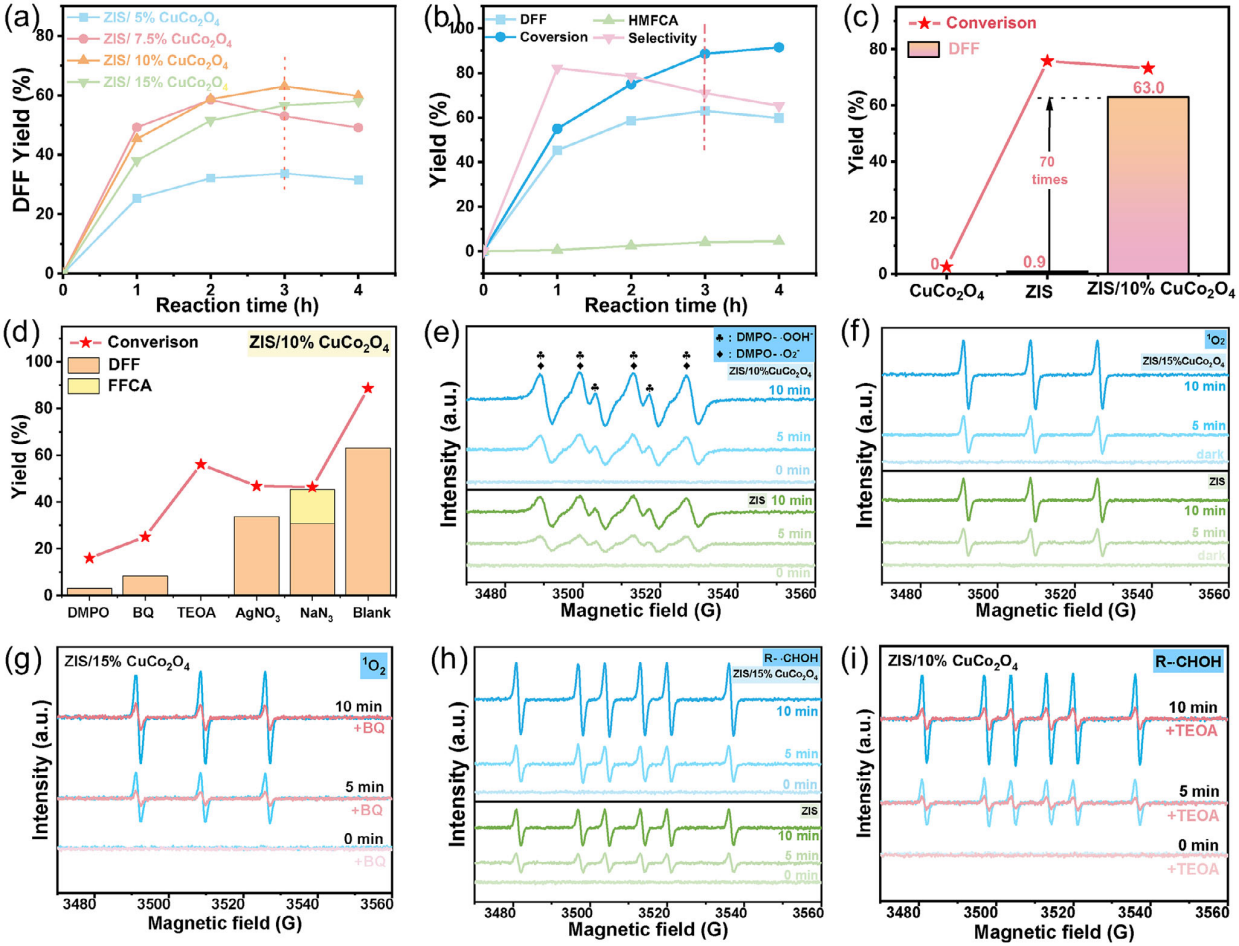
Influence of the a) CuCo_2_O_4_ amount, b) reaction time. c) enhancement of the optimal catalyst compared to ZIS/10%CuCo_2_O_4_ catalyst. d) trapping experiment of HMF to DFF via ZIS/10%CuCo_2_O_4_. In situ ESR spectrum of various radical e) ·OOH and ·O_2_^−^, f) ^1^O_2,_ g) BQ‐^1^O_2_, h) R‐·CHOH, i) TEOA‐R‐·CHOH during light irradiation over ZIS and ZIS/10%CuCo_2_O_4_.

The conversion of HMF in water was subsequently conducted under irradiation with a blue LED. The reaction necessitated light irradiation and catalysts for its progression (Table S2, Supporting Information). When ZIS was used as the catalyst, it exhibited high activity but poor selectivity, with most of the HMF undergoing overoxidation and degradation, resulting in a DFF yield of only 0.9%. In contrast, CuCo_2_O_4_ alone showed minimal activity for the reaction, as HMF could not be converted. Remarkably, when combined, the ZIS/CuCo_2_O_4_ catalyst significantly improved the yield of DFF. As depicted in **Figure** **5**a, the catalysts with varying amounts of CuCo_2_O_4_ exhibited distinct photocatalytic activity in the oxidation of HMF. Regulation of the CuCo_2_O_4_ amounts had been demonstrated to enhance the evolution of HMF conversion to DFF, particularly with the optimal proportion of ZIS/10%CuCo_2_O_4_, yielding twice the DFF rate of ZIS/5%CuCo_2_O_4_ within the first hour. Prolonging the reaction time leads to the over‐oxidation of a small amount of DFF to 5‐formyl‐2‐furancarboxylic acid (FFCA) and 2, 5‐furandicarboxylic acid (FDCA), resulting in a decreased yield of DFF. The transformation path is shown in Figure S6 (Supporting Information). Notably, the ZIS/10%CuCo_2_O_4_ demonstrated exceptional performance, achieving a remarkable 88.6% conversion of HMF within 3 h in the air while maintaining a 71% DFF selectivity (Figure 5b; FigureS7, Supporting Information), the DFF yield was substantially enhanced by 70 times under optimized conditions by ZIS/10%CuCo_2_O_4_ (Figure 5c). It is noteworthy that during the first hour of the reaction, the evolution of DFF reaches 724.9 µmol·g^−1^·h^−1^, surpassing the efficiency of most reported photocatalytic aerobic oxidation methods for HMF (< 300 µmol·g^−1^·h^−1^). The iodometric method does not detect the generation of H_2_O_2_, potentially due to its rapid decomposition in water. The influence of the atmosphere was studied (Figure S7, Supporting Information), and it demonstrated that O_2_ from air is essential and sufficient for the oxidation process of HMF to DFF. The pH influence was studied as well cause acidic conditions favor reduction reactions, while alkaline environments are conducive to oxidation reactions. Under acidic conditions (pH = 4), the conversion of HMF is significantly inhibited, achieving only 54.5%. The yield of DFF is 35.5%, and the yield of FFCA is merely 0.8%. This is attributed to the fact that acidic conditions are more favorable for reduction reactions rather than oxidation reactions. In an alkaline environment with pH = 11, after 3 h of reaction, we observed an HMF conversion rate of only 41.3%, and the product distribution was 1.0% DFF, 1.4% 5‐hydroxymethyl‐2‐furancarboxylic acid (HMFCA), and 3.0% FDCA. Most of the HMF was mineralized to H_2_O_2_ and CO_2_, and DFF was over‐oxidized to FFCA under alkaline conditions, corroborating previously reported findings.^[^ ^25^ ^]^ On the one hand, OH⁻ competes with the ─CHOH group of HMF, resulting in a decreased conversion rate of HMF in alkaline conditions. On the other hand, the enhanced presence of radicals can lead to the mineralization of HMF in strongly alkaline environments, thereby preventing the formation of the desired product DFF.^[^ ^26^ ^]^

The influence of different quenchers on the activity of ZIS and ZIS/10%CuCo_2_O_4_ were shown in Figure S8 (Supporting Information) and Figure 5d.  A significant reduction of the conversion for HMF was observed with the addition of 5,5‐Dimethyl‐1‐pyrroline‐N‐oxide (DMPO), suggesting this process underwent a radical path. AgNO_3_, triethanolamine (TEOA), p‐benzoquinone (BQ), and NaN_3_ selectively inhibited the generation of e^−^, h^+^, and superoxide radicals (·O_2_^−^) and singlet oxygen (^1^O_2_). In the reaction catalyzed by ZIS, since the substrates were almost completely degraded, capturing excessively strong free radicals would be beneficial in mitigating their degradation, resulting in small amounts of DFF and FFCA. For ZIS/10%CuCo_2_O_4_, BQ and AgNO_3_ significantly reduced the conversion rate of HMF (25.1% and 32.3%), with DFF yield decreasing to 8.3% and 19.9%. A similar decline in catalytic activity suggested that ·O_2_^−^ is the main active species generated by light irradiation, confirming that e^−^ was primarily used to reduce O_2_ to ·O_2_^−^ under light excitation. NaN_3,_ as a scavenger for ^1^O_2_, had a minimal impact on the reaction activity compared to other active species (·O_2_^−^, e^−,^ and h^+^). This was because it did not affect the initial activation of O_2_ and the generation of ·O_2_^−^, with ^1^O_2_ only formed after ·O_2_^−^ was oxidized by h^+^ and subsequently participated in later reactions. The addition of TEOA led to no detectable products, confirming the key role of photogenerated h^+^ in the oxidation process. These results preliminarily indicated that e^−^, h^+^, ·O_2_^−^, and ^1^O_2_ act synergistically in the photocatalytic conversion of HMF to DFF.

It is well‐documented that ZIS‐based photocatalysts struggle to maintain stability due to photo‐corrosion, a challenge that is particularly exacerbated when in aqueous rather than organic solvents. The used catalyst was directly retrieved by centrifugation due to its heterogeneous nature, followed by drying for subsequent application in the oxidation of HMF. It was observed that the catalytic efficiency of the reused ZIS/10%CuCo_2_O_4_ significantly declined compared to its initial operation, indicating inevitable photocorrosion during the photocatalytic process. Specifically, after three runs in water, the catalyst exhibited a decrease in HMF conversion rate from 88.6% to 32.7% and 19.9%, and the DFF yield declined from 63.0% to 23.1% and 8.6% (Figure S9, Supporting Information). Similarly, Zhao et al. reported that the HMF conversion rate of ZIS with (110)/(102) facets dropped to ≈26.8% during the second run in acetonitrile.^[^ ^27^ ^]^ The post‐reaction solution analysis via Inductively Coupled Plasma (ICP) revealed leaching of In^3+^ and Zn^2+^ at concentrations of 10.58 and 139.16 mg L^−1^, respectively, suggesting that photodegradation primarily disrupts Zn─S bonds in the ZIS crystal rather than In─S bonds.^[^ ^28^ ^]^ As for CuCo_2_O_4_, the Co^3+^ and Cu^2+^ leachings were 49.04 and 0.31 mg L^−1^, respectively, Cu^2+^ demonstrated remarkable stability within the system.

Electron spin resonance (ESR) analyses were employed to reveal the active species during the reaction. As shown in Figure 5e, a clear typical quadruple peak represents ⋅O_2_^−^ signal, which was observed under light irradiation for both ZIS and ZIS/10%CuCo_2_O_4_.^[^ ^29^ ^]^⋅O_2_^−^ can be easily converted into ·OOH radical by capturing one proton (⋅O_2_^−^ + H^+^ →·OOH, a reversible process in aqueous solution). Generally, ⋅O_2_^−^is characterized by a quartet ESR signal of equal intensity. However, as shown in Figure 5, an in situ ESR measurement detected a sextet signal, which is typically attributed to the protonation of ⋅O_2_^−^ resulting in ·OOH radicals.^[^ ^30^ ^]^ The triple peak in Figure 5f shows the intensity of ^1^O_2_, while Figure 5g illustrates the significant decrease in ^1^O_2_ content upon capturing ·O_2_^−^ with BQ, indicating that ^1^O_2_ was generated from ·O_2_^−^. The six peaks in Figure 5h represent R‐·CHOH, it was observed that the peak intensity did not decrease after trapping ^1^O_2_ with NaN_3_ (Figure S10, Supporting Information), but decreased significantly after trapping h^+^ with TEOA (Figure 5i), suggesting that the R‐·CHOH radical was mainly produced by the direct attack of the C─H bond by h^+^ rather than ^1^O_2_. All active species (·O_2_^−^, ^1^O_2_, and R‐·CHOH) intensities of ZIS/10%CuCo_2_O_4_ were ≈2 times higher than those of ZIS, which intuitively explained the enhancing surface reaction of ZIS/10%CuCo_2_O_4_ and confirmed the improved active species in the entire reaction by establishing a cascade electric field. The results of the capture experiments mutually corroborated the conclusions above, demonstrating the transformations of O_2_ and the formation pathway of R‐·CHOH.

**Figure 6:**
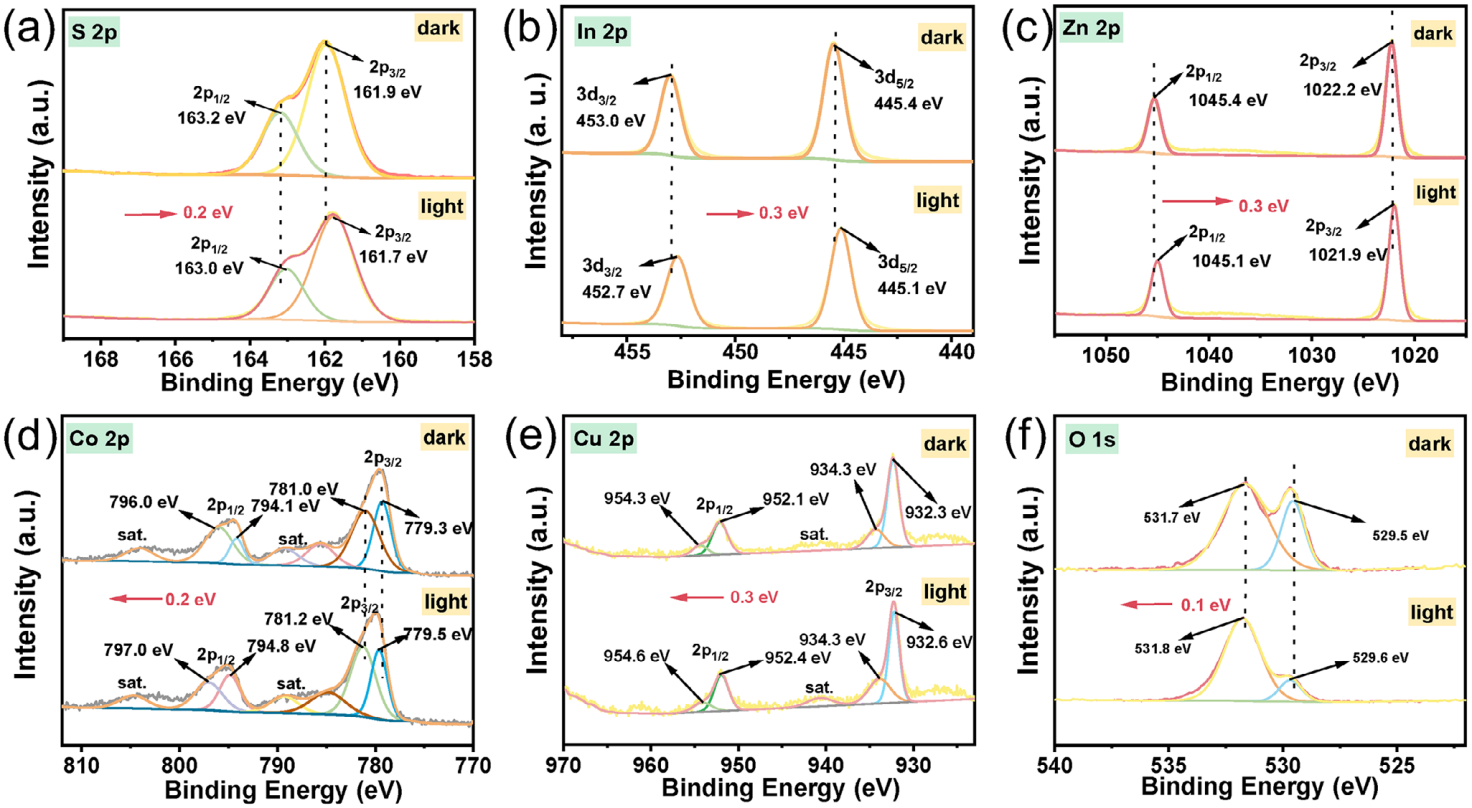
In situ XPS spectra of a) S 2p, b) In 3d, c) Zn 2p, d) Co 2p, e) Cu 2p, f) O 1s of ZIS/10%CuCo_2_O_4_ catalyst under dark and light conditions.

The results here demonstrated the effectiveness of establishing a strong cascaded electric field through B‐IEF and I‐IEF constructed via cocatalyst CuCo_2_O_4_ to modulate charge transfer pathways for efficient biomass conversion. Additionally, the coupling of O_2_ activation by e^−^ and HMF oxidation by hole‐induced deprotonation provided an efficient transfer carrier for photogenerated h^+^ and e^−^. Therefore, this highly efficient photocatalytic oxidation benefited from the cooperation of cascaded IEF to achieve spatial charge separation and accelerate h^+^ transfer to enhance reaction kinetics. In situ X‐ray photoelectron spectroscopy (XPS) can directly elucidate charge transfer by analyzing the shifts in binding energies. The changes in binding energy result from the transfer of photogenerated electrons during irradiation, where an increase or decrease in binding energy corresponds to the loss or gain of electrons, respectively. Post‐illumination, the binding energy peaks of S 2p, In 3d, and Zn 2p exhibited shifts toward higher values (**Figure** **6**a–c), whereas the peaks of Co 2p, Cu 2p, and O 1s moved toward lower binding energies (Figure 6d–f). This phenomenon indicated that the h^+^ should transfer from ZIS to CuCo_2_O_4_, which is consistent with the SPV and band analysis results in Figure 3h. The increase in e^−^ to ZIS upon illumination is primarily attributed to the confinement and accumulation of ZIS photogenerated e^−^ at the CB with high reduction potential, a result of the presence of energy barriers. It well explains why the composite material ZIS/10%CuCo_2_O_4_ can suppress the mineralization of ZIS in HMF conversion.

## 3 Mechanism Study for the Integrated System

**Figure 7:**
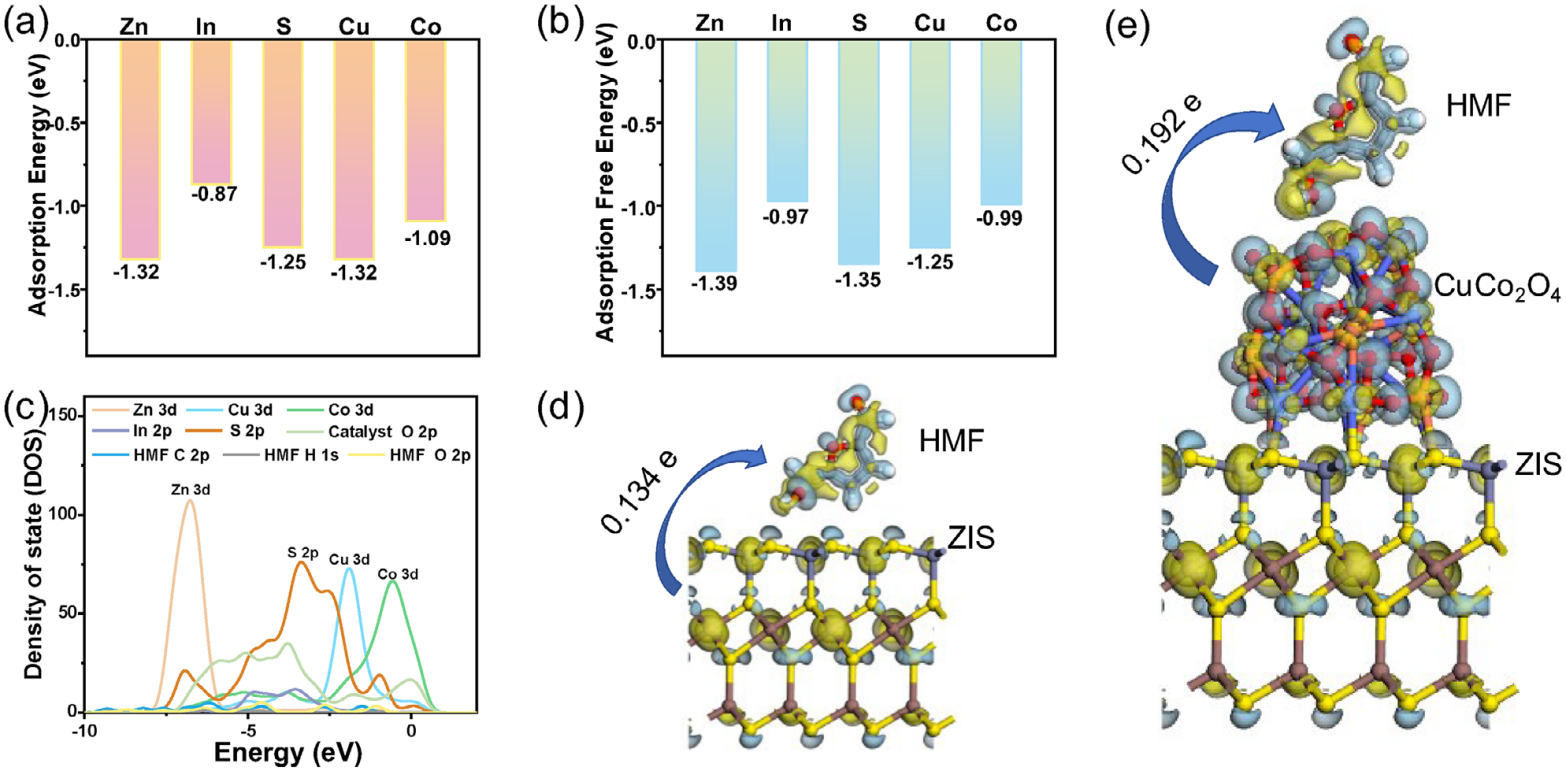
DFT calculation of the a) adsorption energy and b) adsorption‐free energy for different sites. c) DOS for HMF adsorption in ZIS/CuCo_2_O_4._ Bader charges analysis for HMF adsorption in d) ZIS and e) ZIS/CuCo_2_O_4_.

Density functional theory (DFT) offers profound insights into the reaction mechanisms. The adsorption energies computed for various sites on the heterojunction catalyst, as depicted in **Figure** **7**a, were all found to be less than −0.5 eV. This indicates that the adsorption of HMF is primarily governed by chemisorption rather than van der Waals interactions.^[^ ^31^ ^]^ Figure 7b illustrates the Gibbs free energy of adsorption for HMF at Zn, In, S, Cu, Co, and O sites via the oxygen atom in the ─CH_2_OH group. The results suggest that HMF forms a more stable adsorption structure at Zn, S, and Cu sites compared to In and Co sites, due to the lower adsorption free energy. Specifically, for bare ZIS, HMF is observed to bond spontaneously with Zn and S sites with low adsorption free energies (−1.39 eV for Zn and −1.25 eV for S). Similarly, HMF can be easily anchored on the Cu site of CuCo_2_O_4_, with an adsorption free energy of −1.25 eV. These findings indicate that Zn‐S and Cu serve as active sites for HMF conversion, facilitating efficient electron transfer through the Zn─S─Cu interfacial channel, consistent with previous discussions. Furthermore, Figure 7c presents the partial density of states (PDOS) of the ZIS/CuCo_2_O_4_ composite post‐HMF adsorption. The significant overlap between S 2p and Cu 3d orbitals suggests the formation of new chemical bonds at the composite interface, corroborating previous XPS analysis. In the case of HMF, the overlap of C 2p and O 2p orbitals with the substrate atom orbitals indicates preferential activation of the C─OH bond in the hydroxymethyl group of HMF. Additionally, the Bader charge analysis shown in Figure 7d,e reveals that pristine ZIS donates 0.134 electrons upon HMF adsorption, whereas CuCo_2_O_4_ transfers 0.192 electrons to HMF. This suggests that HMF is more readily activated on the CuCo_2_O_4_ component of the ZIS/CuCo_2_O_4_ heterojunction.

**Figure 8:**
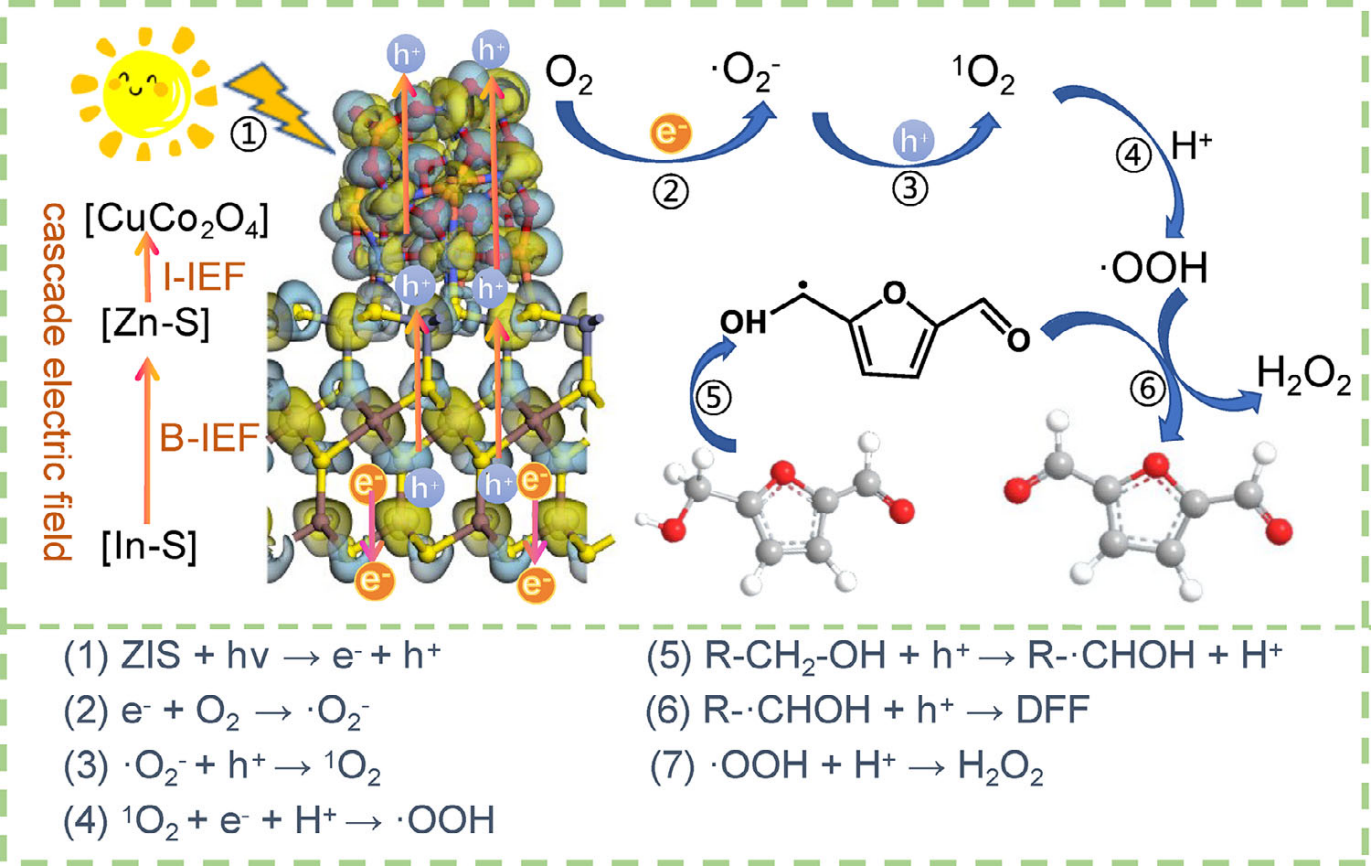
Possible reaction mechanism of HMF to DFF via ZIS/10%CuCo_2_O_4_ catalyst.

Based on the above results, the reaction mechanism for the enhanced photocatalytic performance of ZIS/10%CuCo_2_O_4_ for the conversion of HMF to DFF was proposed. Typically, HMF was anchored to the surface of the ZIS/10%CuCo_2_O_4_ catalyst through the O atom in the alcohol hydroxyl group, and the e^−^ was transferred from CuCo_2_O_4_ to HMF, which needed to undergo two dehydrogenation steps to obtain DFF under the synergistic effect of cascade electric fields and h^+^ transfer. The cleavage of the C─H bond took place on the catalyst surface, playing a crucial role in the selective oxidation reaction of HMF, and was regarded as the pivotal step in the process.^[^ ^32^ ^]^ **Figure** **8** illustrates the overall reaction process; the photoexcited energetic electron–hole pairs are generated first. Then, the separation of photo‐generated electron–hole pairs was promoted by the B‐IEF within ZIS, which directed photo‐generated holes to migrate from the [In‐S] layer to the [Zn‐S] layer, while photo‐generated electrons spontaneously migrated to the In‐S layer. Subsequently, a well‐defined I‐IEF between the ZIS and CuCo_2_O_4_ interfaces can provide a strong driving force for electron migration. Therefore, through the synergistic action of cascading electric fields, effective charge separation between the bulk and surface of the photocatalyst could be achieved. Then, the e^−^ on the surface of the catalyst can activate O_2_ to produce ·O_2_^‐^, which then reacts with h^+^ to form ^1^O_2_, it combines with the extracted H atom from the C─H bond and forms ·OOH. Ultimately, the ·OOH continued to extract an H atom via the R‐·CHOH radical, yielding the value‐added product DFF and the byproduct H_2_O_2_.^[^ ^27^ ^]^

## 4 Oxidation of Other Alcohols Catalyzed by ZIS/10%CuCo_2_O_4_

Based on the above experimental and characterization evidence, it has been demonstrated that ZIS/10%CuCo_2_O_4_ was effective for the alcohol oxidation of the important platform molecule HMF derived from biomass. This success motivated further exploration of other similar alcohol transformations on ZIS/10%CuCo_2_O_4_. The results indicated that ZIS/10%CuCo_2_O_4_, with its cascading electric field energy and high charge separation efficiency, exhibits outstanding photocatalytic performance and selectivity for 11 other heterocyclic and aromatic alcohols. As shown in **Table** **1**, ZIS/10%CuCo_2_O_4_ displayed nearly 100% selectivity to the corresponding aldehydes for 8 tested substrates, including the biomass‐derived 4‐methoxyphenylpropanol and DL‐1‐phenylethanol, 2‐pyridinemethanol, 2‐thiophenemethanol, benzyl alcohol, and para‐substituted benzyl alcohols. Furfuryl alcohol, p‐nitrobenzyl alcohol, and p‐methylbenzyl alcohol exhibited over 90% selectivity to the corresponding aldehydes. However, ZIS/10%CuCo_2_O_4_ showed poor catalytic activity toward linear alcohols such as n‐butanol and n‐hexanol, possibly due to steric hindrance caused by the hydrophobic carbon chains in these alcohols. It is noteworthy that, traditionally, most reactions of this kind require organic solvents such as ACN for catalysis. However, this catalyst has shown the potential to perform alcohol oxidation reactions in water without any additives. Experimental results using different substrates verified the broad potential application of ZIS/10%CuCo_2_O_4_.

**Table 1:** Alcohols oxidation catalyzed by ZIS/10%CuCo_2_O_4_ catalyst.

Substrates	Products	Yield [%]	Selectivity [%]
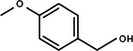	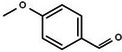	93.4	98.2
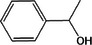	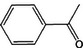	78.0	>99
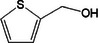	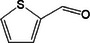	40.9	92.1
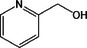	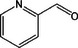	84.5	>99
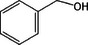	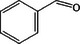	92.9	>99
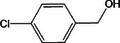	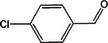	>99	>99
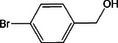	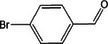	79.2	>99
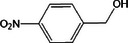	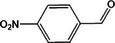	>99	>99
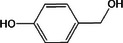	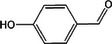	45.0	48.5
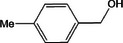	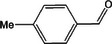	59.9	79.1
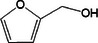	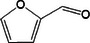	41.2	50.5

## 5 Conclusion

In conclusion, by constructing an outstanding cascading electric field within the photocatalyst and utilizing efficient hole transfer carriers during the reaction, we have successfully achieved the oxidation of biomass‐derived HMF in water using a ZIS‐based photocatalyst. The ZIS/10%CuCo_2_O_4_ heterojunction not only retained the high conversion of HMF but also increased the DFF yield by 70 times (724.9 µmol·g^−1^·h^−1^). The direction of electron flow after illumination was visually demonstrated through characterizations such as KPFM and SPV. DFT calculations and experimental observations also indicated that the cascading electric fields of B‐IEF and I‐IEF can effectively accelerate the charge separation process in bulk and on the surface while suppressing field losses. Therefore, through these synergistic modifications, the overall charge utilization efficiency was optimized, leading to enhanced photocatalytic performance. This study provides a new synergistic strategy to overcome the limitations of slow charge dynamics and slow hole transfer in photocatalytic oxidation processes. Substrate expansion also confirmed the potential of ZIS‐based semiconductors as efficient polarized photocatalysts, which can be developed for green solvent‐based photocatalytic alcohol oxidation reactions.

## 6 Experimental Section

### Sample Preparation—Preparation of ZIS

Specifically, a solution was prepared by dispersing 1 mmol Zn(Ac)_2_∙2H_2_O and 2 mmol InCl_3_∙4H_2_O in 50 mL deionized (DI) water. Subsequently, 4 mmol CH_3_CSNH_2_ (TAA) was introduced incrementally and stirred for 30 min. Then, the solution was transferred to a hermetically sealed reaction vessel and subjected to thermal treatment at 160 °C for 12 h. After cooling to ambient temperature, the resulting precipitates were subjected to triple wash cycles with DI water and ultimately dried at 60 °C for 6 h. The resultant orange powder, post‐grinding, represents the intended catalyst.

### Sample Preparation—Preparation of CuCo_2_O_4_

Initially, 4 mmol Cu(NO)_3_, 8 mmol Co(NO)_3_, and 15 mmol urea were added to a beaker. After stirring in 60 mL DI water for 20 min, the mixture was transferred to a three‐necked flask and heated at 100 °C for 1.5 h. Then (NH_4_)_2_CO_3_ aqueous solution was added slowly in the flask. The product was centrifuged, dried for 8 h, and ground into powder. The resulting powder was then calcined at 350 °C in the muffle furnace in the air for 2 h, followed by grinding to obtain CuCo_2_O_4_.

### Sample Preparation—Preparation of ZIS/CuCo_2_O_4_

Weighing 100 mg of ZIS and varying amounts of CuCo_2_O_4_ (5, 7.5, 10, 15 mg) were separately placed into two beakers and treated with 10 mL of ethanol under ultrasonication for 3 min. After mixing, the combined solution underwent an additional 3 min of ultrasonication before being transferred to a heatable magnetic stirrer. The mixture was then stirred at 300 rpm and heated at 60 °C overnight, resulting in a powdered form denoted as the ZnIn_2_S_4_/CuCo_2_O_4_ catalyst. The color of the catalyst transitioned from light green to progressively darker shades of emerald green with increasing amounts of CuCo_2_O_4_.

### Photocatalytic HMF Oxidation

The photocatalytic oxidation of HMF was conducted using a photochemical reactor fitted with a 445 ± 10 nm LED light source (20 W). Typically, 20 mg of catalyst was dispersed in a 30 mL quartz tube containing 10 mL of deionized (DI) water with an initial HMF concentration of 3 mm. The suspension was subjected to air atmosphere during the reaction, and samples were collected at specific time intervals. HMF, DFF, FFCA, and HMFCA were quantified using high‐performance liquid chromatography (HPLC) equipped with DAD at a wavelength of 248 nm. The generated H_2_O_2_ was quantified using a reagent color‐developing method. Further details on the characterization methods and calculation equations can be found in the Supporting Information.

## Conflict of Interest

The authors declare no conflict of interest.

## Author Contributions

Y.L. performed visualization, methodology, investigation, formal analysis, and wrote the original draft. W.X. performed resources, methodology, investigation, wrote reviewed and edited the final manuscript. J.Y. performed conceptualization. R.Z. performed validation. A.P.R. performed resources. J.Z. performed supervision, project administration, methodology, investigation, wrote reviewed and edited the final manuscript.

## Supporting information



Supporting Information

## Data Availability

The data that support the findings of this study are available in the supplementary material of this article.
