# Supplementary material for: Cascade Electric Field in ZnIn2S4/CuCo2O4 Photocatalyst for the Selective Oxidation of Biomass‐Derived 5‐Hydroxymethylfurfural in Aqueous Solutions
Source: Small. 2025 Mar 19;21(17):2409005. doi: 10.1002/smll.202409005 (PMC12036556; doi:10.1002/smll.202409005)
Supplement: Supplementary file 1 — Supporting Information [file SMLL-21-2409005-s001.docx]

**Supplementary Materials**

**Cascade Electric Field in ZnIn_2_S_4_/CuCo_2_O_4_ Photocatalyst for the Selective Oxidation of Biomass-Derived 5-Hydroxymethylfurfural in Aqueous Solutions**

*Yixuan Liu, Wenhua Xue^*^, Jian Ye, Ruilong Zhang, Akkammagari Putta Rangappa, Jun Zhao ^*^*

Department of Biology, Hong Kong Baptist University, Kowloon Tong, Hong Kong SAR, China.

Corresponding author: [xuewenhua@hkbu.edu.hk](mailto:xuewenhua@hkbu.edu.hk) (W. Xue) , [zhaojun@hkbu.edu.hk](mailto:zhaojun@hkbu.edu.hk) (J. Zhao)

**1. Part I: Experimental Section**

**1.1 Materials**

All the reagents are purchased without further purification. The water used in experiments is self-made ultrapure water with an electrical resistivity of 18 MΩ‧cm. Acetonitrile (ACN) (HPLC grade, water content < 30 ppm) is obtained from Fisher Chemical (USA). DMF (AR, 99.5%, water content < 0.1%) and Dimethyl sulfoxide (DMSO) (AR, 99%, water content < 0.2 %), anhydrous acetonitrile (water content < 10 ppm), CH_3_CSNH_2_ (TAA), InCl_3_∙4H_2_O, Zn(AC)_3_·6H_2_O, 5,5-dimethyl-1-pyrroline N-oxide (DMPO), oxalic acid (AO), Benzoquinone (BQ), sodium azide (NaN_3_), Butylated hydroxytoluene (BHT), HMF (98%), DFF, FFCA, H_2_O_2_, benzyl alcohol and its derivatives, 2-Pyridinemethanol, 2-Thiophenemethanol and Cyclohexanol et al. are all purchased from Aladdin Bio-Chem Technology Co., LTD, Shanghai.

**1.2 Physical characterization**

X-ray diffractometer (XRD, Rigaku Smartlab) with Cu Kα radiation (λ = 0.154 nm) was used to determine the crystal structure of samples. Energy dispersive spectrum (EDS) and transmission electron microscopy (Tecnai G2 F30) were collected to analyze the morphology of the photocatalyst. The UV/vis spectrophotometer (Shimadzu UV-3600) was employed to analyze the photoresponse properties of the catalysts. In order to further study the chemical composition of samples, X-ray photoelectron spectroscopy (XPS, Kratos AXIS NOVA spectrometer) was performed. The Brunauer-Emmett-Teller (BET) method was used to determine the specific surface area.

**1.3 Photocatalytic** **HMF oxidation**

The photocatalytic HMF oxidation was carried out via a photochemical reactor equipped with 445 ± 10 nm LED (20 W). Typically, 20 mg catalyst was dispersed in a 15 mL quartz tube containing 10 mL H_2_O with HMF concentration of 5 mM (unless otherwise noted). After ultrasonic treatment for 5 min, the suspension is employed under air atmosphere for reaction, and sampling after a certain reaction time. For a different atmosphere, the air in the reactor was extracted first by a vacuum pump, and then N_2_ or O_2_ was injected.

For other alcohol substrates, the procedures are the same as above HMF oxidation excepting add different feedstocks in the reaction solution of 10 mL DI water. Subsequently, the reaction temperature and catalyst dosage were optimized to maximize the HMF conversion, DFF yield, and selectivity, all of which were calculated using equations 1-3.

$HMF Conversion (\%)=\frac{\mathrm{HMF}_{I}-\mathrm{HMF}_{F}}{\mathrm{HMF}_{I}}\times1$00 (1)

$DFF Yield (\%)=\frac{\mathrm{DFF}}{\mathrm{HMF}_{I}}\times1$00 (2)

$DFF Selectivity (\%)=\frac{DFF Yield (\%)}{\mathrm{HMF}_{I}}\times1$00 (3)

Where HMFI is the initial HMF concentration and HMFF is the concentration of HMF after the reaction.

**1.4 Products quantification**

High-performance liquid chromatography (HPLC) on a WATERS 2695 with an ultraviolet-visible detector was employed for quantification of HMF, DFF, FFCA, and FDCA (Wavelength: 248 nm. Column: Bio-Rad, Aminex HXP-87P. Mobile phase: 5 mM H_2_SO_4_. Flow rate: 0.6 mL/min). For other alcohol substrates, Agilent 7890A equipped with an FID detector and gas chromatograph-mass spectrometer (GC-MS, GC 7890A/5975C inert XL MSD) were used for quantification. The possible gas product through the photocatalytic process was quantified by gas chromatography (Agilent 7890 equipped with a Molecular Sieve packed column, and a thermal conductivity detector). The identification and quantification of the products were determined via the calibration curves by applying standard solutions with known concentrations of commercially purchased pure reactants, intermediates, and products.

The produced H_2_O_2_ was quantified by the reagent color-developing method. First, 1 mL filtered reaction solution was attenuated by 2 mL DI water, after that, 1 mL of the attenuated sample was added into a mixed solution of 1 mL KI (0.4 M) aqueous solution and 1 mL commercial pH buffer with pH=4 (potassium biphthalate). Finally, the solution was kept for 1 h in t dark. The H_2_O_2_ concentration can be determined by the triiodide anions (I^3-^) concentration according to the following equation, where the I^3-^ can be estimated by Lambert-Beer's law due to their strong absorbance at 350 nm.

H_2_O_2_+3I^-^ + 2H+→I^3-^+2H_2_O

Scheme 1. Calibration curve of H_2_O_2._

**1.5 Quenching Experiment**

The quenching experiments of active species were conducted by adding extra radical scavengers (general condition: 10 mg/30 uL scavengers in 10 mL reaction solution) to investigate the role of radicals during photocatalytic HMF selective oxidation to DFF. 5,5-dimethyl-1-pyrroline-N-oxide (DMPO) was employed as a radical scavenger. Benzoquinone (BQ), NaN_3_, oxalic acid (H_2_C_2_O_4_), and Butylated hydroxytoluene (BHT) were added as scavengers for superoxide radicals, singlet oxygen, H_2_O_2_, and carbon central radical, respectively.

**1.6 Photochemical characterization**

To prepare the working electrodes, 5.0 mg catalyst was dispersed in 15 mL deionized water and then sonicated for 20 min. Subsequently, 1 mL of the solution was dropped homogeneously on a 2 cm × 3 cm fluoride-doped tin oxide (FTO) glass. After drying at ambient temperature, the FTO working electrodes were used without further processing.

**1.7 DFT calculation**

DFT calculations were made using DMol3 for geometry optimization and property analysis.[1] The Perdew–Burke–Ernzerhof (PBE) function with van der Waals (vdW) interactions proposed by Grimme (DFT-D2) is adopted to represent electron-ion interactions.[2] The generalized gradient approximation (GGA) pseudopotential3 is utilized to represent electron exchange and associated effects.[3] The 3 × 3 × 1 supercell structure was adopted to construct the slab surface, meanwhile the 20 Å-thick vacuum was added into the models to avoid the interactions between periodic images. An orbital cutoff of 5.6 Å was used for all computations. Convergence was considered achieved when the energy reached a threshold of 1.0 × 10^−5^ Ha, the forces reached 0.002 Ha Å^−1^, and the displacements reached 0.005 Å. Brillouin zone sampling was performed by using the Monkhost-Pack scheme with a k-point grid of 4 × 4 × 1.

**2. Part II: Supplementary Results**

**Table S1** Performance of current studies on photocatalytic HMF to DFF in water

| Catalysts (mg) | HMF(mM) | Time(h) | Conv.(%) | Yield.(%) | Refs. |
| --- | --- | --- | --- | --- | --- |
| TiO_2_ (30 mg) | 0.5 | 16 | 50 | 13 | [4] |
| Cu_2_O/TiO_2_ (30 mg) | 1 | 1.5 | 27.1 | 23 | [5] |
| Cu-porphyrin/g-C_3_N_4_ (50 mg) | 0.5 | 4 | 72 | 27.3 | [6] |
| WO_3_/C_3_N_4_ | 0.1 | 6 | 27 | 23.5 | [7] |
| Exfoliated g-C_3_N_4_ | 0.5 | 4 | 40 | 20 | [8] |
| Pd/g-C_3_N_4_ (20 mg) | 0.5 | 5 | 67 | 28.7 | [9] |
| g-C_3_N_4_ | 0.5 | 4 | 100 | 25 | [10] |
| g-C_3_N_4_ | 0.5 | 4 | 69 | 27.9 | [11] |
| Zn-Porphyrin/g-C_3_N_4_(pilot plant reactor) | 0.5 | 4 | 73 | 26.3 | [12] |
| g-C_3_N_4_-H_2_O_2_ adduct | 0.5 | 4 | 47 | 33.3 | [13] |
| g-C_3_N_4_-H_2_O_2_ (pilot plant reactor) | 0.5 | 4 | 25 | 23.1 | [14] |
| WO_3_/g-C_3_N_4_ | 0.1 | - | 32.1 | 8.5 | [15] |
| ZIS/CuCo_2_O_4_(20 mg) | 3 | 3 | 88.6 | 63.0 | This work |

**Table S2** Photocatalytic conversion of HMF to DFF under different conditions

| Entry | Photocatalyst | Light | HMF Con. (%) | DFF Yield (%) | DFF Sel. (%) |
| --- | --- | --- | --- | --- | --- |
| 1 | No | LED (445 nm) | 0 | 0 | 0 |
| 2 | ZIS | LED (445 nm) | 91.7 | 0.9 | 1 |
| 3 | CuCo_2_O_4_ | LED (445 nm) | 3.1 | 0 | 0 |
| 4 | ZIS/10%CuCo_2_O_4_ | LED (445 nm) | 88.6 | 63 | 71 |
| 5 | ZIS/10%CuCo_2_O_4_ | No | 0 | 0 | 0 |

Reaction conditions: 3 mM HMF, 10 mL water, 20 mg catalyst loading, air, 20 °C, 3h

\

**Figure S1.** SEM images of a, b) ZIS c) CuCo_2_O_4_ d) ZIS/10%CuCo_2_O_4._


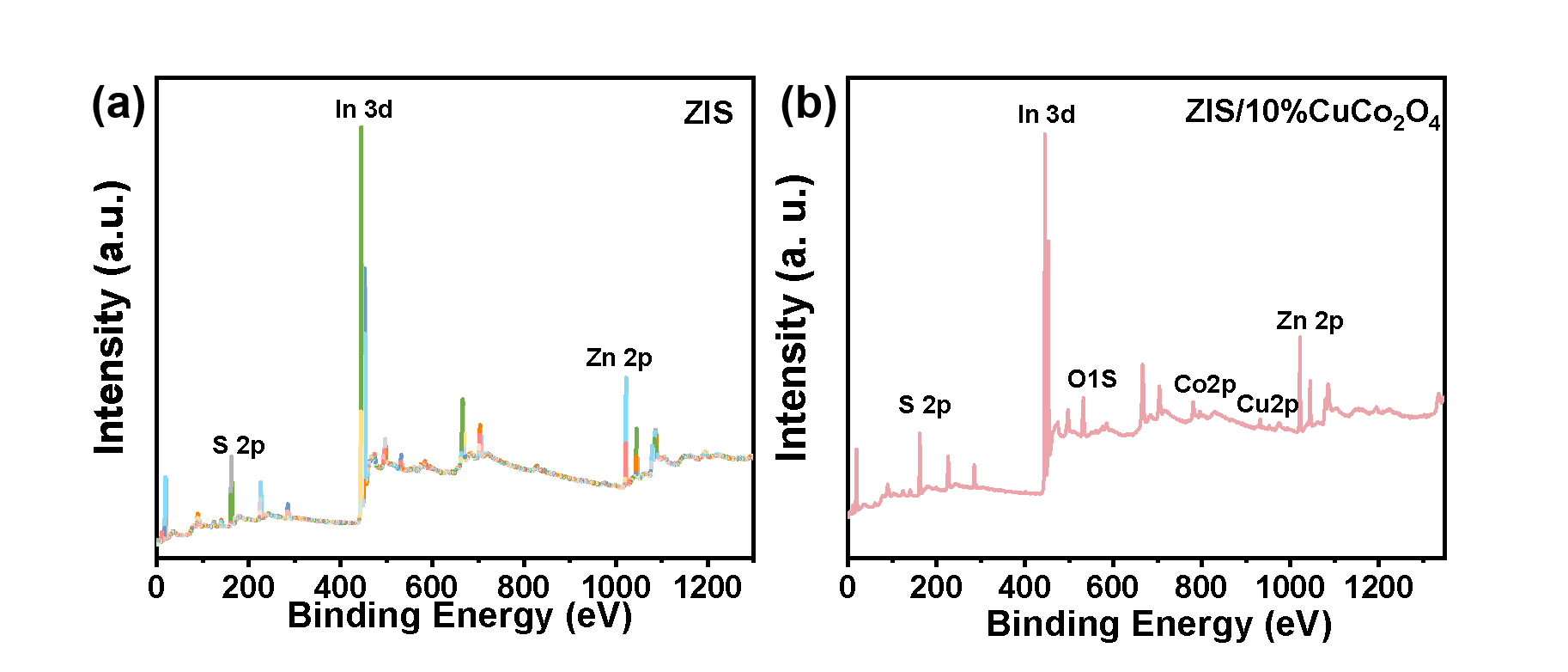


**Figure S2.** XPS survey spectra of (a) ZIS (b) ZIS/10%CuCo_2_O_4_


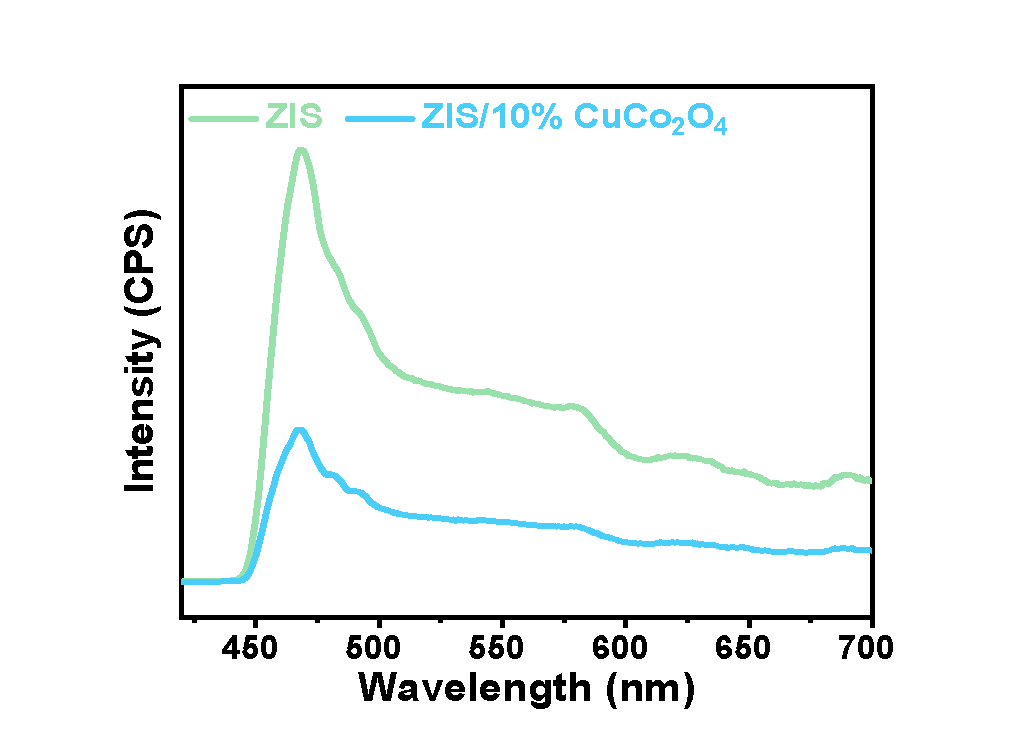


**Figure S3.** Steady-state PL spectra of ZIS and ZIS/10%CuCo_2_O_4_


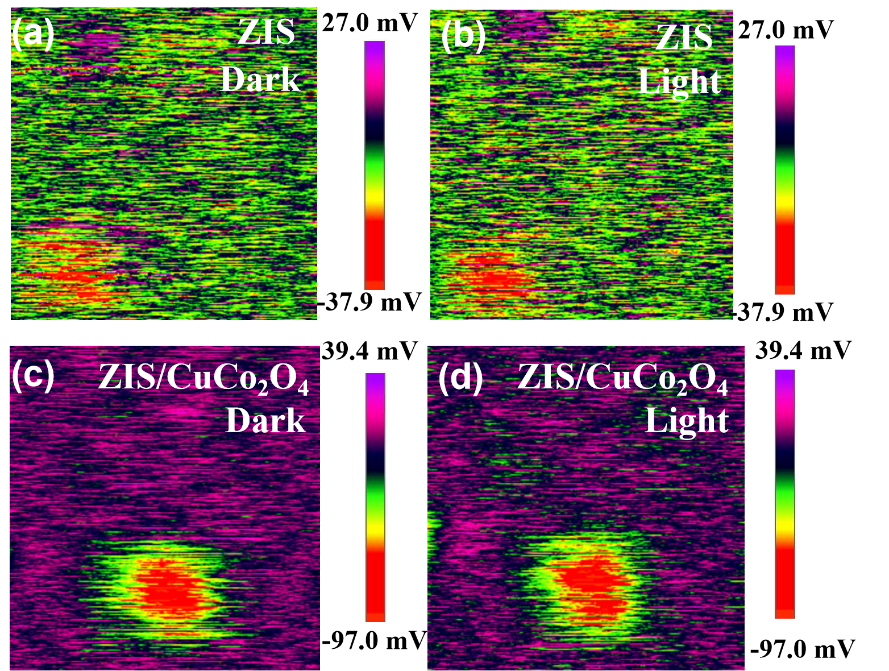


**Figure S4.** KPFM potential 2D images along the line in the dark (a, c) and under illumination (b, d).


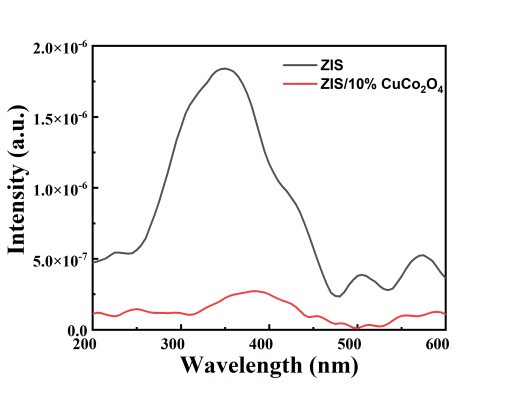


**Figure S5.** SPV spectra of ZIS and ZIS/10%CuCo_2_O_4_.


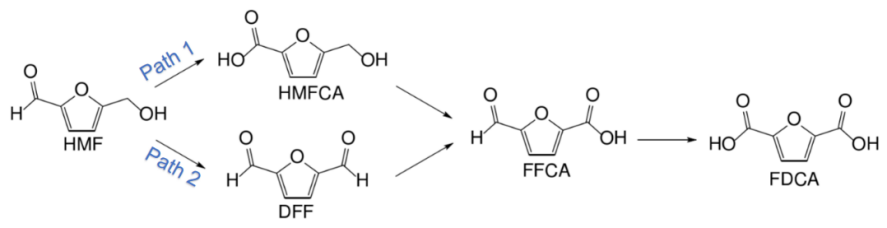


**Figure S6.** Schematic diagram of HMF oxidation path.

**Figure S7.** Influence of reaction atmosphere.

Reaction conditions: 3 mM HMF, 10 mL water, 20 mg catalyst loading, 20 °C.


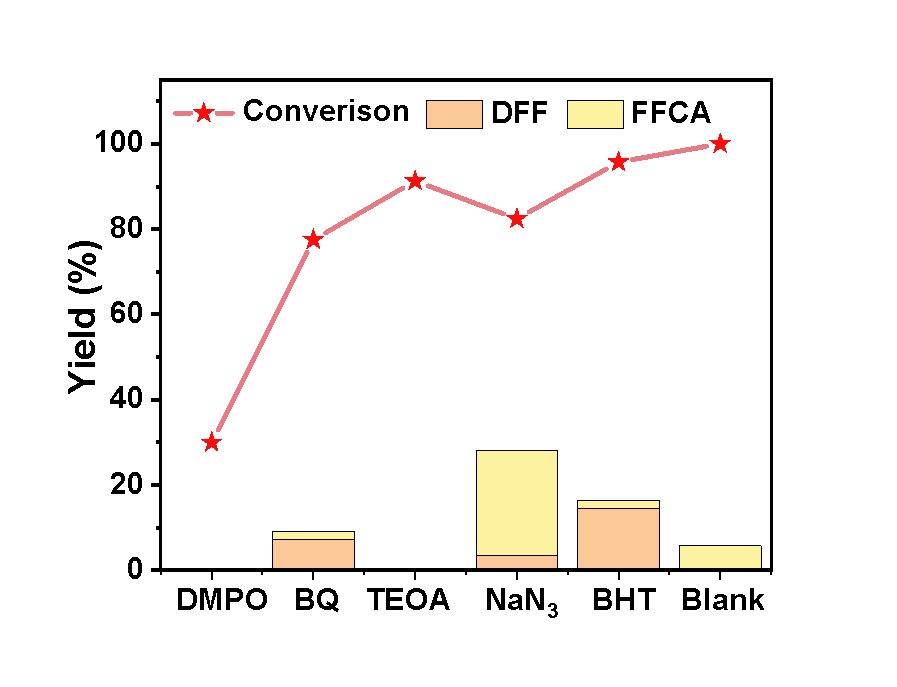


**Figure S8.** Trapping experiment of HMF to DFF via ZIS

Reaction conditions: 3 mM HMF, 10 mL water, 20 mg catalyst loading, 20 °C in air.


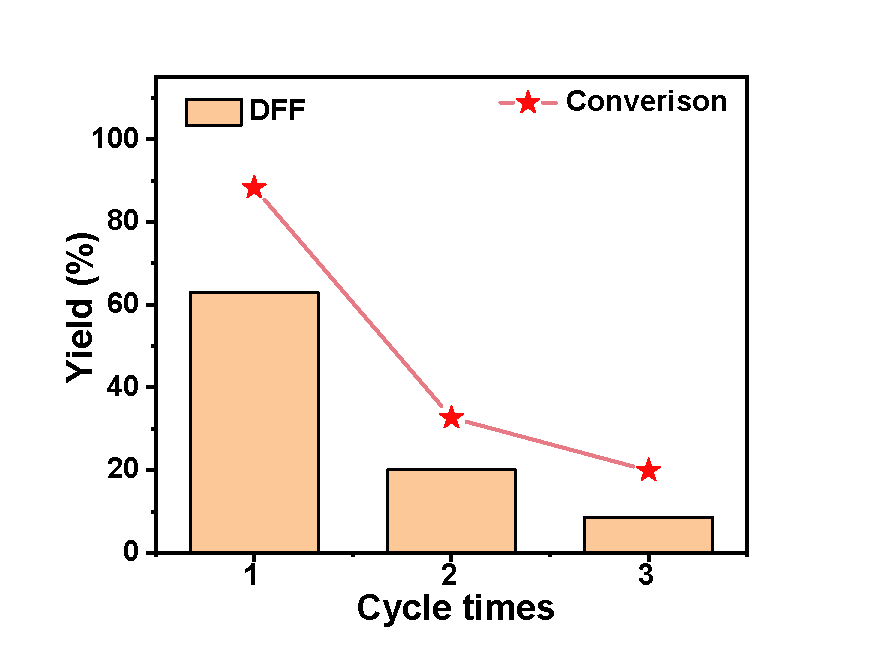


**Figure S9.** Recyclability of ZIS/10%CuCo_2_O_4_ for HMF oxidation

Reaction conditions: 3 mM HMF, 10 mL water, 20 mg catalyst loading, 20 °C in air.


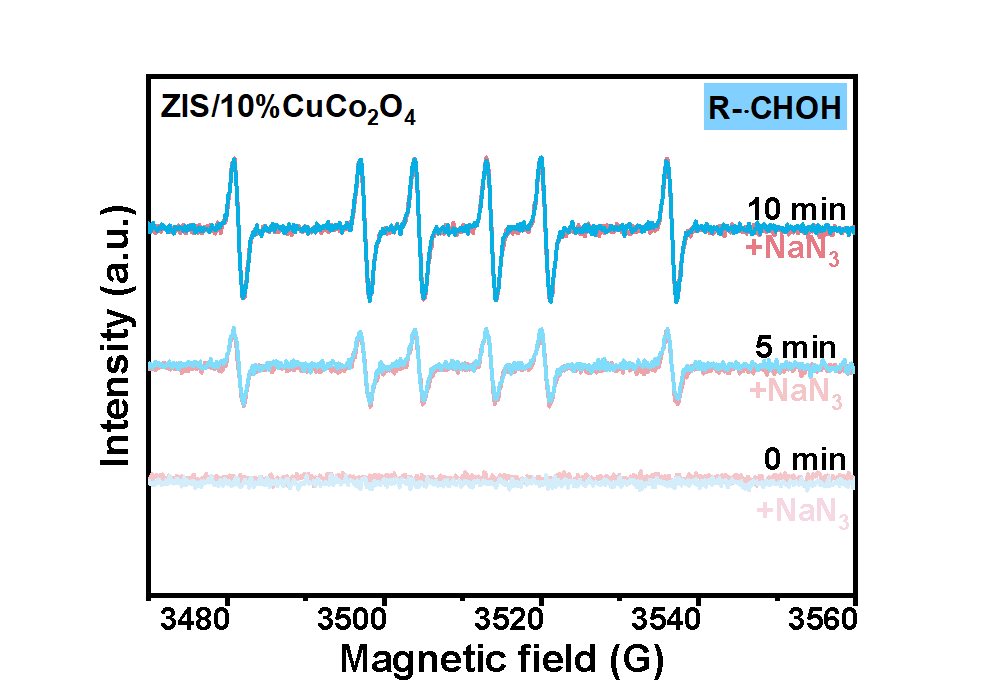


**Figure S10.** In-situ ESR spectrum of NaN_3_-R-·CHOH radical during light irradiation over ZIS/10%CuCo_2_O_4_.

**References**

1. B. Delley, *J. Chem. Phys*. **1990**, *92*, 508-517.
2. S. Grimme, Inc. *J. Comput. Chem.* **2004**, *25*, 1463–1473.
3. S. Grimme, *J. Comput. Chem.* **2006**, *27,* 1787-99.
4. S. Yurdakal, B. S. Tek, O. Alagöz, V. Augugliaro, V. Loddo, G. Palmisano, L. Palmisano, *ACS Sustainable Chem. Eng.* **2013**, *1*, 456-461.
5. M. Zhang, Y. Zhang, L. Ye, Z. Yu, R. Liu, Y. Qiao, L. Sun, J. Cui, X. Lu, *Appl. Catal. B: Environ.* **2023**, *330*, 122635.
6. E.I. García-López, F. R. Pomilla, E. Bloise, X. Lü, G. Mele, L. Palmisano, G. Marcì, *Top Catal.* **2021**, *64*, 758–771.
7. H. Zhang, Z. Feng, Y. Zhu, Y. Wu, T. Wu, *J. Photochem. Photobiol. A Chem.* **2019**, *371*, 1–9.
8. I. Krivtsov, E.I. García-lópez, G. Marcì, L. Palmisano, E. Díaz, Z. Amghouz, J.R.García, S. Ordóñez, E. Díaz, *Appl. Catal. B Environ.* **2017**, *204*, 430–439.
9. A. E. ElMetwally, M. S. Sayed, Y. Zhou, J. B. Domena, J. Shim, R. M. Leblanc, M. R. Knecht, L. G. Bachas. *J. Phys. Chem.* C **2022,** *126*, 15671–15684.
10. E. I. García‑López1, F. Arcidiacono1, A. D. Vincenzo, L. Palmisano, P. L. Meo, G. Marcì, *Photoch. Photobio. Sci.* **2023**, *22*, 1517-1526.
11. I. Krivtsov, E. I. García-López, G. Marcì, L. Palmisano, Z. Amghouz, J. R. García, S. Ordónez, E. Díaz, *Appl. Catal. B: Environ.* **2017**, *204*, 430–439.
12. E. I. García‑López1, F. R. Pomilla, E. Bloise, X. Lü, G. Mele, L. Palmisano, G. Marc, *Top Catal.* **2021,** *64*, 758–771.
13. M. Ilkaeva, I. Krivtsov, J. R. G., E. Díaz, S. Ordóñez, E. I. García-López, G. Marcì, L. Palmisano, M. I. Maldonado, S. Malato, *Catal. Today*. **2018**, *315*, 138-148.
14. M. Ilkaeva, I. Krivtsov, E. I. García-López, G. Marcì, O. Khainakova, J. R. García, L. Palmisano, E. Díaz, S. Ordóñez, *J. Catal.* **2018,** *359*, 212–222.
15. H. Zhang, Z. Feng, Y. Zhu, Y. Wu, T. Wu, *J. Photochem. Photobiol. A Chem.* **2019**, *371*, 1-9.
